# Disparities in diabetes-related avoidable hospitalization among diabetes patients with disability using a nationwide cohort study

**DOI:** 10.1038/s41598-022-05557-5

**Published:** 2022-02-02

**Authors:** Hin Moi Youn, Dong-Woo Choi, Sung-In Jang, Eun-Cheol Park

**Affiliations:** 1 Department of Family Medican and Primary Care, University of Hong Kong, Hong Kong, China; 2 Present Address: Data Link & Operation Team, Cancer Big Data Center, National Cancer Control Institute, National Cancer Center, Goyang, Republic of Korea; 3 Institute of Health Services Research, Yonsei University, Seoul, Republic of Korea; 4 Department of Preventive Medicine, Yonsei University College of Medicine, Seoul, Republic of Korea

**Keywords:** Diabetes, Disease prevention, Health policy, Public health

## Abstract

Diabetes is an ambulatory care sensitive condition that quality of care can prevent complications development and hospitalization needs. However, diabetes patients with disability face greater challenges with receiving quality diabetes care than those without disabilities. This study examined diabetes-related avoidable hospitalizations (DRAH) focusing on the association with disability. We used nationally representative health insurance cohort data from 2002 to 2013. The study population is people who were newly diagnosed with type 2 diabetes. We measured the cumulated number of DRAH using the Prevention Quality Indicators (PQIs). The variables of interest were disability severity and type. We performed a recurrent events analysis using Cox proportional hazard regression model. Among 49,410 type 2 diabetes patients, 12,231 (24.8%) experienced DRAHs at least once during the follow-up period. Among the total population, 5924 (12.0%) diabetes patients were registered as disabled. The findings report that disability severity was significantly associated with higher risks for DRAH, where severely disabled diabetes patients showed the highest hazard ratio of 2.24 (95% CI 1.80–2.79). Among three DRAH indicators, severely disabled diabetes patients showed increased risks for long-term (AHR 2.21, 95% CI 1.89–2.60) and uncontrolled (AHR 2.28, 95% CI 1.80–2.88) DRAH. In addition, intellectual (AHR 5.52, 95% CI 3.78–8.05) and mental (AHR 3.97, 95% CI 2.29–6.89) disability showed higher risks than other types of disability. In conclusion, diabetes patients with disability are at higher risk for DRAH compared to those without disabilities, and those with intellectual and mental disabilities were more likely to experience DRAH compared to those with physical or other types of disability. These findings call for action to find the more appropriate interventions to improve targeted diabetes primary care for patients with disability. Further research is needed to better understand determinants of increasing risks of DRAH.

## Introduction

Diabetes is a common chronic disease and has become a major global public issue. The global prevalence of diabetes reached to 423 million in 2019, and it is projected to rise even further^1^. It is a leading cause of serious complications and premature deaths among adults aged 20–79 years, with significant health and social costs. Diabetes affects vulnerable populations disproportionately, including those with disabilities, who have higher risks of diabetes and comorbidities compared to the general population^2,3^. In Korea, 19.1% of people with disabilities were diagnosed with diabetes compared to 11.1% in people without disabilities, along with higher rate of obesity which is a key contributing factor to diabetes^4^. Previous studies have found that physical, mental, and intellectual disabilities or limitations increase the risk of developing diabetes and secondary conditions^2,3,5,6^. They may be exposed to greater risks due to a lack of ability to maintain healthy diet and lifestyle, as well as access preventive care. In addition, diabetes management can become more complicated by their limited function, increasing health risks and costs. Given the rising burden, disparities in diabetes became an important concern for health policy and management. The Korean government has made policy efforts to reduce this burden through better diabetes management by focusing on improving access to and quality of primary care for people who need better assistance. For example, ‘program of the physician in charge of health of persons with disability’ was introduced, assigning a primary physician to a person with disabilities, thereby providing continuous and comprehensive care for chronic illnesses such as diabetes^7^.

Diabetes is one of ambulatory care sensitive conditions (ACSC), a set of conditions for which timely and effective care can potentially prevent complications and avoidable hospitalizations (AH)^8^. Diabetes-related avoidable hospitalization (DRAH) is a widely used indicator of the quality of primary diabetes care; thus, high rates of DRAHs imply a lack of access or inadequacy of care^9–11^. Prior studies have demonstrated that hospitalization rates significantly increased with disabilities or limitations^2,12,13^. People with disabilities often have poorer health outcomes due to their underlying conditions, but also limited access to appropriate health care^14,15^. From a public health perspective, disabilities can pose greater challenges to disease management and a higher rate of DRAH among diabetes patients with disabilities can be an important indicator to determine the quality of care. In addition, disparities in DRAH can have health, social, and economic implications. Therefore, assessing the quality of diabetes care could help to recognize their greater needs and to explore ways of minimizing risks within the health care setting.

In the study, we hypothesized that diabetes patients with disability have a higher risk for DRAH compared to those without disability. The purpose of the study is to expand existing literature on DRAH by examining the association between disability and DRAH among diabetes patients.

## Results

Table 1 summarizes the baseline characteristics of the study population. Among 49,410 type 2 diabetes patients, 12,231 (24.8%) experienced DRAHs at least once during the follow-up period. Among the total population, 5924 (12.0%) diabetes patients were registered as disabled including 4577 (9.3%) mild to moderately disabled and 1347 (2.7%) severely patients. The mean age of non-disabled diabetes patients was between 50 to 55 years old, 47.8% (n = 20,768) were male, the mean follow-up years were 5.6 years, and the incidence rate was 24.1% (n = 32,991). Among mild to moderately disabled diabetes patients, the mean age was between 60 to 65 years old, 56.7% (n = 2593) were male, the mean follow-up years were 4.8 years, and the incidence rate was 27.5%. Among severely disabled diabetes patients, the mean age was between 56 to 59 years old, 58.4% (n = 786) were male, the mean follow-up years were 4.4 years, and the incidence rate was 35.5%. We grouped disability type into 7 categories where 3,367 (6.8%) were registered for physical disability and 313 (0.7%) were registered for mental and intellectual disability. Intellectually disabled diabetes patients showed the highest incidence of DRAH (51.9%). There were 24,147 (48.9%) male and 25,263 (51.1%) female diabetes patients and 24,905 (54.5%) were older than 60 years. The proportion of diabetes patients with CCI and DCSI scores greater than 4 were 27,351 (55.4%) and 11,707 (23.7%), respectively.

**Table 1:** Baseline characteristics of the study population.

Variables	Diabetes-related avoidable hospitalization (DRAH)						
	Total		Yes		No		p value
	N	%	N	%	N	%	
	49,410	100.0	12,231	24.8	37,179	75.2	
**Disability**							< 0.0001
Not disabled	43,486	88.0	10,495	24.1	32,991	75.9	
Mild/moderate	4577	9.3	1258	27.5	3319	72.5	
Severe	1347	2.7	478	35.5	869	64.5	
**Type of disability**							< 0.0001
Not disabled	43,486	88.0	10,495	24.1	32,991	75.9	
Physical	3367	6.8	889	26.4	2478	73.6	
Brain lesions	763	1.5	254	33.3	509	66.7	
Hearing	625	1.3	202	32.3	423	67.7	
Visual	602	1.2	167	27.7	435	72.3	
Intellectual	185	0.4	96	51.9	89	48.1	
Mental	128	0.3	60	46.9	68	53.1	
Others^a^	254	0.5	68	26.8	186	73.2	
**Sex**							< 0.0001
Male	24,147	48.9	6630	27.5	17,517	72.5	
Female	25,263	51.1	5601	22.2	19,662	77.8	
**Age**							< 0.0001
20–29	1188	2.4	244	20.5	944	79.5	
30–39	3434	7.0	783	22.8	2651	77.2	
40–49	7917	16.0	2064	26.1	5853	73.9	
50–59	11,966	24.2	3079	25.7	8887	74.3	
60–69	12,724	25.8	3007	23.6	9717	76.4	
70 ≤	12,181	24.7	3054	25.1	9127	74.9	
**Region**							0.0144
Metropolitan	19,846	40.2	4778	24.1	15,068	75.9	
Urban	12,231	24.8	3064	25.1	9167	74.9	
Rural	17,333	35.1	4389	25.3	12,944	74.7	
**Household income**							< 0.0001
Q1 (low)	9846	19.9	2754	28.0	7092	72.0	
Q2	6980	14.1	1702	24.4	5278	75.6	
Q3	8177	16.5	2062	25.2	6115	74.8	
Q4	10,253	20.8	2446	23.9	7807	76.1	
Q5 (high)	14,154	28.6	3267	23.1	10,887	76.9	
**Heath insurance type**							< 0.0001
NHI	46,569	94.3	11,195	24.0	35,374	76.0	
Medical aid	2841	5.7	1036	36.5	1805	63.5	
**CCI**							< 0.0001
0	2083	4.2	589	28.3	1494	71.7	
1	4786	9.7	1121	23.4	3665	76.6	
2	7168	14.5	1625	22.7	5543	77.3	
3	8022	16.2	1713	21.4	6309	78.6	
4 ≤	27,351	55.4	7183	26.3	20,168	73.7	
**DCSI score**							< 0.0001
0	13,695	27.7	3176	23.2	10,519	76.8	
1	9801	19.8	2066	21.1	7735	78.9	
2	8277	16.8	1972	23.8	6305	76.2	
3	5930	12.0	1464	24.7	4466	75.3	
4 ≤	11,707	23.7	3553	30.3	8154	69.7	
**Diagnosis year**							< 0.0001
2004	7185	14.5	1844	25.7	5341	74.3	
2005	7245	14.7	1620	22.4	5625	77.6	
2006	5802	11.7	1315	22.7	4487	77.3	
2007	5633	11.4	1322	23.5	4311	76.5	
2008	5713	11.6	1496	26.2	4217	73.8	
2009	4858	9.8	1213	25.0	3645	75.0	
2010	3995	8.1	981	24.6	3014	75.4	
2011	4177	8.5	1088	26.0	3089	74.0	
2012	2965	6.0	771	26.0	2194	74.0	
2013	1837	3.7	581	31.6	1256	68.4	

*NHI:* National Health Insurance, *CCI:* Charlson Comorbidity Index, *DCSI:* Diabetes Complications Severity Index. ^a^Others include autistic disorder, cardiac dysfunction, respiratory dysfunction, hepatic dysfunction, facial disfigurement, intestinal fistula and urinary fistula, epilepsy.

**Figure 1:**
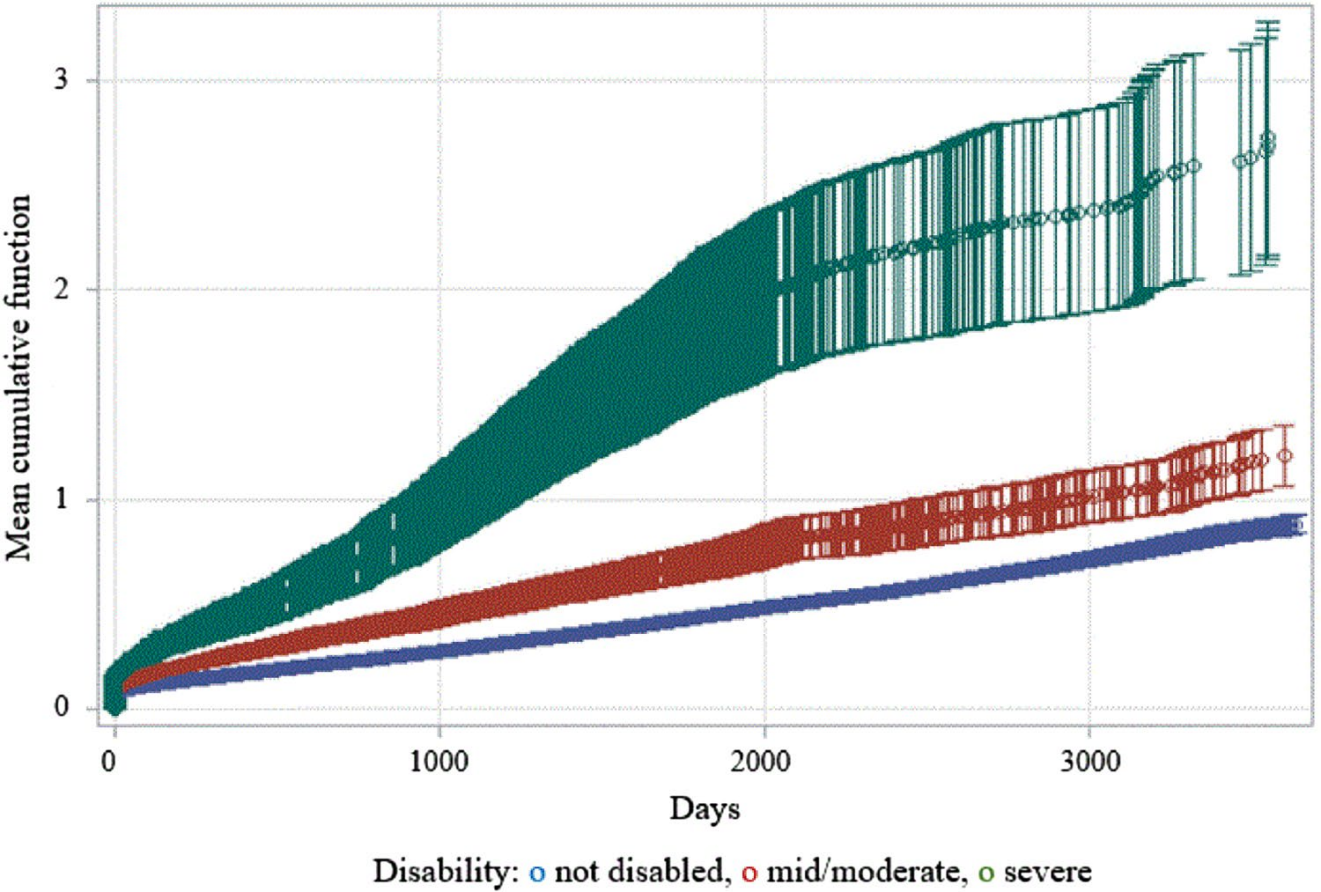
Cumulative mean function estimates for diabetes-related avoidable hospitalization according to disability severity.

Figure 1 presents the mean cumulative function curves showing that diabetes patients with severe disability had higher cumulative number of recurrences of DRAH.

The association between disability with DRAH was evaluated using a recurrent event survival analysis. Table 2 shows that disability was associated with an increased hazard ratio for DRAH, where severely disabled diabetes patients had the greatest hazard ratio of 2.24 (95% CI 1.80–2.79). Diabetes patients with higher risk for DRAH were male, older age, living in non-metropolitan areas, and Medicaid beneficiaries. The findings also present diabetes patients with severe diabetes complications have a higher risk for DRAH (AHR 1.24, 95% CI 1.09–1.41).

**Table 2:** Diabetes-related avoidable hospitalization hazard ratio, results of Cox proportional hazard model.

Variables	Diabetes-related avoidable hospitalization (DRAH)	
	AHR	95% CI
**Disability**		
Not disabled	1.00	
Mild/moderate	1.10	(0.96–1.26)
Severe	2.24	(1.80–2.79)
**Sex**		
Male	1.00	
Female	0.83	(0.77–0.89)
**Age**		
20–29	1.00	
30–39	1.25	(0.97–1.62)
40–49	1.41	(1.11–1.79)
50–59	1.49	(1.17–1.90)
60–69	1.32	(1.04–1.68)
70 ≤	2.06	(1.61–2.65)
**Region**		
Metropolitan	1.00	
Urban	1.17	(1.06–1.29)
Rural	1.14	(1.05–1.25)
**Household income**		
Quantile 1 (low)	1.00	
Quantile 2	1.00	(0.88–1.14)
Quantile 3	0.98	(0.86–1.12)
Quantile 4	0.94	(0.81–1.07)
Quantile 5 (high)	0.90	(0.79–1.02)
**Heath insurance type**		
NHI	1.00	
Medical aid	2.91	(2.46–3.44)
**CCI**		
0	1.00	
1	0.89	(0.64–1.24)
2	0.86	(0.63–1.16)
3	0.76	(0.56–1.03)
4 ≤	1.13	(0.84–1.53)
**DCSI score**		
0	1.00	
1	0.93	(0.81–1.07)
2	1.05	(0.91–1.20)
3	1.05	(0.90–1.22)
4 ≤	1.24	(1.09–1.41)

*AHR* Adjusted Hazard Ratio, *NHI* National Health Insurance, *CCI* Charlson Comorbidity Index, *DCSI* Diabetes Complications Severity Index.

Table 3 shows the results of analysis by DRAH indicators including short-term complications, long-term complications, and uncontrolled diabetes without complications. We found that severe disability is significantly associated with long-term (AHR 2.21, 95% CI 1.89–2.60) and uncontrolled (AHR 2.28, 95% CI 1.80–2.88) DRAH.

**Table 3:** Diabetes-related avoidable hospitalization hazard ratio by different indicators, results of Cox proportional hazard model.

Variables	Diabetes-related avoidable hospitalization (DRAH)					
	Short-term^a^		Long-term^b^		Uncontrolled^c^	
	AHR	95% CI	AHR	95% CI	AHR	95% CI
**Disability**						
Not disabled	1.00		1.00		1.00	
Mild/moderate	0.55	(0.24–1.25)	1.05	(0.91–1.20)	1.12	(0.96–1.29)
Severe	0.72	(0.24–2.18)	2.21	(1.89–2.60)	2.28	(1.80–2.88)

*AHR* Adjusted Hazard Ratio. Adjusted for sex, age, region, income, insurance type, CCI, DCSI score, Year, ^a^short-term DRAH include E10.0, E10.1, E11.1, E13.0, E13.1, E14.0. E14.1., ^b^long-term DRAH include E10.2–8, E11.2–8, E13.2–8, E14.2–8., ^c^uncontrolled DRAH includes E10.9, E11.9, E13.9, E14.9.

**Figure 2:**
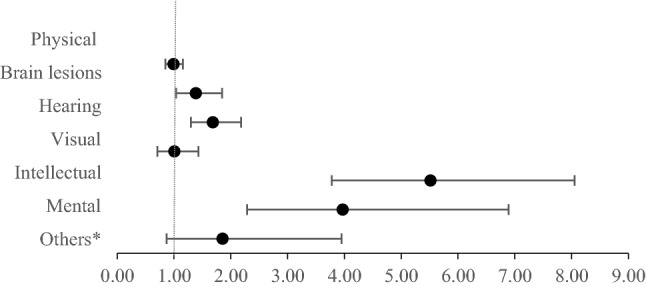
Association between disability types and diabetes-related avoidable hospitalization. *AHR* Adjusted Hazard Ratio. Adjusted for sex, age, region, income, insurance type, CCI, DCSI score, Year * Others include autistic disorder, cardiac dysfunction, respiratory dysfunction, hepatic dysfunction, facial disfigurement, intestinal fistula and urinary fistula, epilepsy.

We further analyzed the association for DRAH according to disability types (Fig. 2). Compared to the non-disabled diabetes patients, those with mental-related disabilities showed higher risks of experiencing DRAH; Intellectual disabilities (AHR 5.52, 95% CI 3.78–8.05), mental health disorders (AHR 3.97, 95% CI 2.29–6.89).

## Discussion

Understanding the relationship between disability and DRAH is an important social and health policy concern, particularly in addressing health disparities. This study aims to find out how disability is associated with DRAH by comparing diabetes patients with disabilities and those without disabilities. We analyzed the risks of DRAH according to severity and types of disability, using a recurrent events analysis model. Our principal finding was that DRAH risks increase for diabetes patients with disability compared to those without disability. We also found that diabetes patients with intellectual disabilities or mental health disorders were more likely to experience DRAH than those with physical or other types of disabilities. Considering diabetes is a widely known ACSC, the findings reveal that diabetes patients with disabilities face greater risks and need more attention for a better quality of disease management and care to reduce health risks and prevent avoidable hospitalizations. Our findings are consistent with previous studies demonstrating that people with disabilities or limitations are particularly vulnerable to avoidable hospitalizations for ACSCs^2,13,16,17^.

Successful diabetes management and strategies require adequately integrated approach of medical care and self-care^18,19^. However, people with disabilities often have the impeded ability of self-care skills and difficulties complying with guidelines, such as keeping healthy behaviors, medication adherence, and medical check-ups^2,13^. Furthermore, they often face more barriers to appropriate healthcare. Frequently reported barriers are financial difficulties, inconvenient transportation, absence of caregiver, and communication problem^20,21^. When diabetes patients with disabilities receive insufficient or inadequate care, it could amplify health and social disparities^14,22^.

The results showed a strong association between age and DRAH, which is noteworthy considering Korea is one of the countries with the most rapidly aging population in the world, and about 46.7% of adults older than 65 years reported having disabilities in 2018^23^. We found increasing risks for DRAH among diabetes patients with lower education, lower income, and Medicaid benefits as well as those living in rural areas. Socioeconomic disparities in DRAH have been proven in earlier studies that low socioeconomic status (SES) is correlated with disability and poor health. People with low SES or living in the disadvantaged areas are more likely to be exposed to unhealthy environments and lower access to essential care^24,25^.

Avoidable hospitalizations for ACSCs are indicators for quality of primary care, which has become a major provider for diabetes management. A crucial role of primary care is to ensure that patients receive the good quality of care that patients need to manage diabetes^18^. In 2018, the Korean government initiated policy intervention for people with disabilities aiming to strengthen the management of chronic diseases and disability conditions by promoting continuous and comprehensive care in primary care settings^7^. People with disabilities require better understanding and communication because of their specific conditions. Thus, strong and productive patient-provider relationships can help to better meet patients’ needs and improve the effectiveness of diabetes management. Recent reports have found some positive responses from providers and patients who participated in the program^26^. However, this pilot program is still in its early stages and the number of participants, both provides and patients, is still not sufficient to evaluate its impact on health outcomes. Future study is necessary to evaluate the program and to provide evidence for policy reforms.

Although this study showed meaningful findings, there are a few limitations that emerged. First, we could not consider individual health-related characteristics and health settings that are correlated to health outcomes for diabetes patients. For instance, we could not control for weight measures such as body mass index (BMI) which is one of the significant predictors for diabetes-related risks^27,28^. In addition, healthy behavior such as dietary habits, smoking, drinking, and excise are important determinants particularly for diabetes^29^. We could not include information on the health settings such as a number of primary care providers. Further research is needed to investigate how healthcare resources or management programs can affect access to care for people with disabilities. Second, this study did not consider patients’ healthcare utilization prior to DRAH occurrence. DRAHs are indicators for quality of primary care, therefore, identifying healthcare utilization including a number of inpatient and outpatient visits, continuity of care, and medication compliance will help better understand disparities in people with disabilities. Third, the results should be interpreted with caution because of potential limitations in the disability registration data. Particularly, the disability grading system which classified disabled people into different groups has been abolished in 2019, in order to enhance the provision of necessary benefits^30^. Fourth, as is true of many secondary data analyses, there may have been reporting errors.

Despite these limitations, this study has contributed to expanding understanding disparities in DRAH among diabetes patients with disability. We put effort into improving methodological rigor. We improved the accuracy of the results by using a nationally representative sample and increased homogeneity by identifying subjects who were newly diagnosed with diabetes during the observation period. We employed a recurrent events analysis using a survival model, which is more suitable to account for multiple occurrences of DRAHs in a subject. Lastly, we adopted validated and widely accepted indicators to measure DRAH.

In conclusion, diabetes patients with disabilities showed higher risks for DRAH compared to those without disabilities, and those with mental and intellectual disabilities were more likely to experience DRAH compared to those with physical disabilities. The findings call for action to find appropriate interventions to improve coordinated and comprehensive primary care and to provide more targeted care for those with disabilities. Further research is needed to better understand determinants of increasing risks of DRAH.

## Materials and methods

### Data source and study population

This was a population-based cohort study analyzing a nationally representative sample of national health insurance subscribers in Korea between 2002 and 2013. The National Health Insurance Service-National Sample Cohort (NHIS-NSC) is comprised 2.2% of the total Korean population enrolled in the NHIS, the single universal insurer in Korea^31^. A total of 1,0254,340 participants were selected through systematic stratified random sampling with proportional allocation within each stratum using the individual’s total annual medical expenses as a target variable for sampling^32^. The data is composed of individual socio-demographic characteristics and all medical claims data including diagnosis codes according to the International Classification of Disease version 10 (ICD-10)^33^. We conducted a cohort study of patients diagnosed with type 2 diabetes mellitus (T2DM)(ICD-10: E11) obtained from medical claims data^34^. We restricted the study samples to patients who were newly diagnosed in order to adjust for potential confounding^35^. We, therefore, identified and excluded patients who have diagnose codes for T2DM during a washout period of two years (2002–2003). Among them, we included diabetes patients who have at least one hospitalization during follow-up period. Furthermore, diabetes patients who were registered for renal disability were excluded to reduce bias from the incidence of hospitalization or death due to underlying conditions^36,37^. Among 1,125,691 participants who were initially enrolled, 126,738 were diagnosed with T2DM during 2002–2013. After excluding those within the washout period (n = 34,807), without any hospitalization (n = 41,815), and with renal disability (n = 308), younger than 19 years old (n = 296), a final analytic sample was 49,411 diabetes patients.

### Variables

An outcome of interest in this study was the occurrence of DRAH. DRAH was measured according to the definition developed by the Agency for Healthcare Research and Quality (AHRQ). We used the Prevention Quality Indicators (PQI), standardized and evidence-based measures of avoidable hospitalizations for ACSCs^38^, which include the three widely used DRAH indicators; diabetes with short-term complications, and diabetes with long-term complications, uncontrolled diabetes without complications^38,39^. First, we used a dichotomous single outcome variable to measure whether or not a patient had DRAH to examine overall experience during follow-up^11,40^. When any DRAH occurs, we coded the event as 1, and otherwise as 0. We then separately analyzed by each type of indicator to account for the differences in the severity of complications.

A variable of interest was the presence of a disability. In this study, we defined disabilities based on the disability registration state. In the dataset, disability is grouped into three according to severity; not-disabled, mild to moderate (grade 1–2), and severe (grade 3–6)^33^. We coded each grade as 0, 1, and 2. For further analysis, we classified patients according to eight disability types: physical disabilities, brain lesions, visual impairment, hearing impairment, intellectual disabilities, mental health disorders, and other disabilities^41^.

We included socio-demographic characteristics including sex (male or female), age (20–29, 30–39, 40–49, 50–59, 60–69, 70–79, or ≥ 80), region (metropolitan, urban, or rural), household income (Q1, Q2, Q3, Q4, or Q5), health insurance type (NHI or Medicaid), and the Charlson’s Comorbidity Index (CCI: 1, 2, or ≥ 3) as a proxy indicator of health status^41^. The Diabetes Complication Severity Index (DCSI) was used to control for the influence by the severity of complications. Diabetes complications include 7 categories (nephropathy, neuropathy, retinopathy, cerebrovascular disease, cardiovascular disease, peripheral vascular disorder, and metabolic diseases). DCSI was based on a scale ranging from 0 to 2 for each complication abnormality, with a total maximum score of 13^42^.

### Statistical analysis

First, baseline characteristics of individuals with DRAH were compared with those without DRAH, using a χ^2^ test. Thereafter, we used extended Cox models to model recurrent time-to-event outcomes to investigate the association between (i) the severity of disability and (ii) the types of disability with DRAH. Recurrent events refer to events of interest experienced more than one time by a given subject. In this study, event of interest is DRAH, which a subject can experience repeatedly during their observation period^43^. We adopted Andersen Gill model (also known as counting process model) which assumes that each event is independent. In this model, a subject would contribute to the risk set for every event as long as the subject is under observation at the time of event occurred, and a subsequent event’s time interval starts at the end of the previous event’s time interval^43–45^. We calculated the adjusted hazard ratio (HR) and 95% CI for DRAH. We analyzed the cumulative mean function estimates for recurrent events data, which we defined as the average number of cumulative events experienced by a given subject at each time point since the beginning of follow-up^46^. In addition, we examined the risks accoridng to each DRAH indicator. All analyses were conducted using SAS software, version 9.3 (SAS Institute, Cary, NC).

### Ethical approval

This study was conducted according to the 2008 Declaration of Helsinki and approved by the independent Institutional Review Board of Yonsei University Health System (IRB number: 2021-1706-001) with no written informed consent because patients’ records/information was anonymized and de-identified prior to analysis. The need of the Informed Consent was waived by the Institutional Review Board of Yonsei University Health System.

## Data Availability

The data that support the findings of this study are available from the National Health Insurance Sharing Service but restrictions apply to the availability of these data.

## Abbreviations

ACSC: Ambulatory care sensitive condition
AH: Avoidable hospitalization
DRAH: Diabetes-related avoidable hospitalization
NHIS: National Health Insurance Service
NHIS-NSC: National Health Insurance Service-National Sample cohort
KCD: Korean Standard Classification of Disease
ICD: International Standard Classification of Disease
AHRD: Agency for Healthcare Research and Quality
PQI: Prevention quality indicator
CCI: Charlson’s Comorbidity Index
DCSI: Diabetes Complication Severity Index
AHR: Adjusted hazard ratio
CI: Confidence interval
SES: Socioeconomic status
